# The gene panel with methylation-related genes and clinical factors for predicting overall survival of endometrial cancer

**DOI:** 10.5937/jomb0-58710

**Published:** 2025-10-28

**Authors:** Lijuan Jiao, Junyan Li, Jiong Ma, Pu Cheng, Yue Pang

**Affiliations:** 1 The Second Affiliated Hospital Zhejiang University School of Medicine, Department of Gynecology, Hangzhou, China; 2 The Second Affiliated Hospital Zhejiang University School of Medicine, Department of Anesthesia Surgery, Hangzhou, China

**Keywords:** endometrial cancer, DNA methylation, biomarker, diagnosis, prognosis, rak endometrijuma, metilacija DNK, bio-marker, dijagnoza, prognoza

## Abstract

**Background:**

This study aimed to identify novel methyla-tion-based prognostic biomarkers for endometrial cancer (EC) to facilitate early diagnosis and treatment. To explore methylation-related prognostic markers in endometrial tis-sue by analyzing TCGA data and to establish a methylation-based risk model for EC patients.

**Methods:**

We systematically analyzed methylation-related gene expression and prognostic significance in 409 EC patients using TCGA DNA methylation data. DNA methy-lation biomarkers were identified through consensus clus-tering and weighted gene co-expression network analysis (WGCNA). The clusterProfiler algorithm was employed to determine key signaling pathways across different sub-groups. A gene panel targeting critical DNA methylation sites was subsequently constructed.

**Results:**

A methylation-related prognostic risk model was developed, incorporating five CpG sites: cg01416891, cg00082235, cg01493517,cg03811891,and cg05317207. The model demonstrated strong predictive performance, with high-risk patients exhibiting significantly poorer prog-noses compared to low-risk patients. A gene panel was also established to predict prognosis across different EC risk groups.

**Conclusions:**

The methylation-related gene panel model serves as a reliable prognostic biomarker for EC, offering potential for enhanced early diagnosis and personalized treatment strategies.

## Abstract (sr)

**Uvod:**

Ova studija je imala za cilj da identifikuje nove prog-nostičke biomarkere za rak endometrijuma (EC) zasnovane na metilaciji kako bi se olakšila rana dijagnoza i lečenje. Clj je bio da se istraže prognostički makeri vezani za metilaciju u tkivu endometrijuma analizom TCGA podataka i uspo staviti mo del rizika zasnovan na metilaciji za pacijente sa EC.

**Metode:**

Sistematski smo analizirali ekspresiju gena povezanu sa metilacijom i prognostički značaj kod 409 EC pacijenata koristeći podatke o metilaciji TCGA DNK. Biomarkeri metilacije DNK identifikovani su konsenzusom grupisanjem i analizom mreže koekspresije gena (VGCNA). Algoritam clusterProfiler je korišćen za određivanje ključnih signalnih puteva u različitim podgrupama. Nakon toga je napravljen genski panel koji cilja kritična mesta metilacije DNK.

**Rezultati:**

Razvijen je model prognostičkog rizika koji se odnosi na metilaciju, koji uključuje pet CpG lokacija: cg01416891, cg00082235, cg01493517, cg03811891 i cg05317207. Model je pokazao snažne prediktivne perfor-manse, pri čemu su visokorizični pacijenti pokazali znatno lošiju prognozu u poređenju sa pacijentima sa niskim rizikom. T akođe je uspostavljen genski panel za predviđanje prognoze za različite EC rizične grupe.

**Zaključak:**

Model panela gena koji se odnosi na metilaciju služi kao pouzdan prognostički biomarker za EC, nudeći potencijal za poboljšanu ranu dijagnozu i personalizovane strategije lečenja.

## Introduction

Endometrial cancer (EC) ranks first in the gynecologic cancer incidence in developed countries. It isone of the three major malignant tumors of female genital system, Although 67% of patients exhibit early-stage disease and boast a high 5-year overall survival (OS), it’s important to note that the 5-year OS for EC patients in stage IVA and IVB are less than 20% [1]. Vaginal bleeding is the most usual premonitor of EC. Most affected women can be diagnosed early and enjoy good prognosis.

However, patients diagnosed in advanced stages demonstrate a high recurrence rate and poor prognosis. At present, staging and histological classification of Federation of Gynecology and Obstetrics (FIGO) are the primary tools applied in the stratification and prognosis prediction of patients with EC. Accurate prognosis prediction system is paramount to guide personalized therapies and assess the recurrence risk to improve clinical outcome.

EC is a heterogeneous disease with different genetic, molecular and pathological characteristics.The diagnosis of EC is based on pathological types identified by histology and immunohistochemistry [2]. Transvaginal ultrasonography (TVS) for the assessment of endometrial thickness is widely regarded as the initial approach for identifying EC in females. However, TVS has a poor specificity and sensitivity in EC detection. The identification of unnatural endometrium by cervical smear can assess the pathological degree of the endometrium, although it lacks the sensitivity and accuracy to diagnose EC. The entire accuracy of cervical cytology in EC detection is 38% [3]. Proof of tissue lesion is the gold standard for diagnosing EC. Cervical dilation curettage and hysteroscopy are frequently employed for EC detection. It is reported that sensitivities for pre- and post-menopausal women are 91% and 99.6% respectively [4]. However, endometrial biopsy can cause considerable discomfort to patients [5], and the majority of patients suffer these uncomfortable procedures without cancer. Furthermore, the amount of tissue using these techniques for pathological evaluation is constrained, ranging from 25% to 36% [6]. Consequently, due to the deficiency of a content large-scale endometrium assessment method, a screening protocol for EC women has not yet been established.

Currently, some novel molecules have been identified in EC, including genomic mutation,microsatellite instability, DNA methylation and histone modification [7] [8] [9]. DNA methylation is one of the DNA modifications, which can change the genetic expression without changing the DNA sequence. Researches have shown that DNA methylation control gene expression by change DNA conformation, DNA stability and the interaction between DNA and protein. DNA hypermethylation in gene promoter leads to the epigenetic silencing of tumor suppressor genes, which often serves as an early cancer. Aberrant DNA methylation are closely linked to the development and progression of many cancers. Distinctive DNA methylation signatures have been increasingly utilized in diagnosing a range of cancers, including ovarian carcinoma, breast cancer, prostate cancer, renal cell cancer and EC [6] [10] [11] [12] [13]. Alterations in HS3ST2 and KLF4 gene methylation status play a pivotal role in the development of EC [14]. Increased methylation of the paired box 2 (PAX2) promoter was observed in EC cell lines and tissues, which could increase the ability of cell viability and invasion [15]. Methylation of the SHP1 gene demonstrates a strong specificity for identifying EC, and CDH13 promoter methylation is implicated in early stage of EC [16]. These references indicate that DNA methylation could be as diagnostic biomarkers,which provide targets for noninvasive early diagnosis and monitoring for cancers. In this study, we collected the DNA methylation profiles of EC patients from the Cancer Genome Atlas (TCGA) database and investigated the categorization of EC based on distinct prognostic subtypes. By stratifying patients into subgroups with distinct survival outcomes, this model aims to guide clinical decision-making by: Improving risk stratification to identify high-risk patients who may benefit from adjuvant therapies or closer surveillance; Facilitating personalized treatment selection by linking methylation-based subtypes to therapeutic responsiveness (e.g., immunotherapy, chemotherapy); Enhancing diagnostic precision by integrating methylation biomarkers into routine clinical workflows to complement histopathological assessment. This approach to classification may uncover new EC molecular subtypes to more precisely categorize EC patients. In addition, our categorization framework offers clinicians a new insight in EC diagnosis and personalized treatments.

## Materials and methods

### DNA methylation and RNA data collection from TCGA

DNA methylation information of EC patients was downloaded from the TCGA program database on May 1, 2024 (Version 2.0). For each probe, the hypermethylation status was quantified by the b value, corresponding to fully-methylated and unmethylated. Probes exhibiting missing data in over 75% of the samples were omitted from the subsequent analysis. Moreover, the cross-reactive probes of CpGs were removed according to the list identified by Discovery of cross-reactive probes and polymorphic CpGs. For remaining probes with missing values (NAs), the k-nearest neighbors (knn) imputation method was employed to fill in the gaps. The ComBat algorithm was utilized to eliminate batch effects by consolidating all the DNA methylation data and considering batch and patient ID information [17]. Moreover, unstable genomic sites, including CpGs in sex chromosomes, the cross-reactive probes and polymorphic CpGs were removed. Finally, 208022 CpGs were chosen for further analysis.

RNA-seq and clinical information contain Age, Stage and Grade for 409 EC samples were downloaded from the TCGA. Next, we divided the database into two cohorts by using randomization: validation and training set. Finally, 409 EC samples with DNA methylation profiles and expression profiles were screened for ultimate analysis.

### COX risk regression model

A univariate Cox proportional hazards regression model to assess the association between the methylation levels of individual CpG sites and the hazard of an event, while also considering patient age, estrogen receptor (ER) status, tumor stage, and survival information. This univariate analysis helped us identify potential CpG sites and clinical factors that may be associated with the outcome of interest. To further evaluate the independent effects of these variables and control for potential confounding factors, we then conducted a multivariate Cox proportional hazards regression analysis. The coxph function, available within the survival package in R, was employed to fit the Cox proportional hazards models to the methylation status of CpG sites, while integrating clinical details. For each CpG site, a multivariate Cox proportional hazards regression model formula was established, incorporating the methylation level along with other significant clinical factors identified in the univariate analysis.

### Consensus clustering analysis

To identify distinct subgroups of EC, consensus clustering was conducted using the ConcensusClusterPlus package. In every iteration, a subset comprising 80% of the tumors was selected for analysis. The k-means algorithm method using Euclidean square distance as the similarity quantity was utilized. These robust results were undergone more than 100 iterations. Each sample’d-score was derived based on the following formula:

d = Σk N=1 (x11 - y 11)^2^ + L + (x1k - y 1k)^2^ + L + (x1N - y 1N)^2^

Following the application of the Con sen sus ClusterPlus algorithm, both cluster-level and itemlevel consensus outcomes were generated. The selection of the optimal number of clusters was guided by three primary criteria: ensuring a relatively high degree of internal cluster consistency, maintaining a low coefficient of variation (CV), and observing no significant rise in the area beneath the cumulative distribution function (CDF) curve. The CV was computed using the formula: CV = (SD/MN) *100%, in which MN denotes the average of samples and SD represents the standard deviation.

### Survival and clinical characteristics analysis

To visualize the overall survival trends across different EC subgroups categorized by DNA methylation patterns, Kaplan-Meier survival curves were constructed. The log-rank test was employed to ascertain whether there were statistically significant differences among these subgroups. The survival package in the relevant statistical software was utilized to carry out these survival analyses. Relationship between biological and clinical traits with DNA hypermethylation clusters-based subgroups were analyzed using the chisquare test.

### Specific DNA methylation markers for EC subgroups

In the current investigation, quantitative methodology was adopted to detect the specific DNA methylation CpGs in EC subgroups for identifying quantitative differentially methylated regions (QDMRs). The assessment of DNA methylation disparities across numerous samples and the recognition of sample-specific characteristics are critical components of genomic functional analysis. Regions that exhibit different methylation patterns in numerous samples are considered as potential epigenetic functional areas containing transcriptional regulation. Consequently, the assessment of differentially methylated regions (DMRs) across numerous samples offers a more thorough examination for EC targets. We used a threshold established by QDMR derived from the hypermethylation feasibility model. Additionally, the sample specificity of each DMR can be quantified by QDMR. For each DMR r, the entropy *HQ *signifies the extent of methylation variability in the entire sample. Zhang et al. described the advantages of a specific sample S for the analysis of the entire methylation difference. Therefore, this subgroup has the greatest absolute classification sample specificity.

### Prognosis model based on Bayesian network

A supervised Bayesian network classification model was formulated using the training set and this model can determine specific CpG sites. Subsequently, this model was employed to categorize the samples in the validation set into corresponding subgroups. The calculation formula of the risk score is as follows:

RiskScore=27.825*cg01416891+42.503*cg00082235+34.264*cg01493517+50.001*cg03811891+90.949*cg05317207

### Gene ontology (GO) and Kyoto encyclopedia of genes and genomes (KEGG) analysis

In the study, clusterProfler, org.Hs.eg.db, ggplot2 and enrichplot package were employed to analyze the DEGs and the functions of DEGs between low-risk and high-risk groups. Functional candidate elements that did not meet both criteria (logFC > 1.5 and adjusted P < 0.05) were omitted from further analysis. This combined filtering approach enhances the robustness of our findings by focusing on genes with both substantial differential expression and statistically significant pathway enrichment.

### Co-expression network construction using WGCNA

Prior to network construction, methylation β-values of 8,161 CpGs were preprocessed to exclude outliers. Samples with missing values >10% or abnormal clustering (assessed by hierarchical clustering and principal component analysis) were removed. The weighted adjacency matrix was constructed using a signed network to preserve directionality of correlations, with the soft thresholding power (β) set to 7 based on the scale-free topology criterion. The adjacency matrix was transformed into a Topological Over lap Matrix (TOM) to minimize noise and highlight biologically meaningful interactions. Hierarchical clustering with average link-age and dynamic tree cutting (minimum module size = 30 CpGs, merge cut height = 0.25) identified 12 co-expression modules. The gray module, representing CpGs not assigned to any functional cluster, was excluded from downstream analyses.

### Statistical analysis

R (version 4.2.1) was employed for statistical analysis. A Student’s t-test was conducted to compare the expression of risk score in distinct groups. K-M survival analysis was used to estimate the fatidic capacity of the prognostic model. *P*<0.05 was considered as statistically significant.

## Results

### DNA methylation cluster analysis

A total of 409 examples were divided into two queues: the training sets (204 samples) and validation sets (205 samples). Each CpG site in training set were constructed a prediction model with survival information of cases. As a result, 29129 CpG sites were significantly related to prognosis (*P* < 0.05). Ultimately, 8161 CpG sites demonstrated statistical significance and served as the final classification features. Then, top 20 methylation sites were showed in Table 1.

**Table 1:** Top 20 methylation sites.

CpGs	p.value	HR	Low 95%CI	High 95%CI
cg20227766	1.62E-08	1214.459	103.3094	14276.64
cg24884961	1.66E-08	0.001367	0.000138	0.013503
cg26974500	2.63E-07	5.73E+13	3.32E+08	9.92E+18
cg27650089	3.16E-07	0.019289	0.004247	0.087605
cg04393701	4.26E-07	0.036268	0.010029	0.131162
cg12057615	5.88E-07	1.73E+27	3.55E+16	8.42E+37
cg14394253	5.90E-07	6.60E+44	1.70E+27	2.56E+62
cg02848157	6.80E-07	0.001126	7.73E-05	0.016406
cg05104283	6.88E-07	0.000447	2.13E-05	0.009394
cg26688472	6.90E-07	19907967	26134.1	1.52E+10
cg26236902	1.35E-06	0.000887	5.13E-05	0.015336
cg12598007	1.39E-06	4.25E+23	1.08E+14	1.67E+33
cg01712359	1.57E-06	0.002329	0.000196	0.027645
cg23371050	1.63E-06	4.59E+10	2011841	1.05E+15
cg15203632	1.68E-06	0.001651	0.00012	0.022728
cg01928143	1.77E-06	1.28E-06	4.91E-09	0.000335
cg12332917	1.81E-06	1.04E+14	1.83E+08	5.91E+19
cg16741710	1.87E-06	1645.587	78.30757	34581.02
cg10525574	1.89E-06	0.000965	5.55E-05	0.016785
cg26705765	1.92E-06	0.008289	0.001153	0.059597

**Figure 1:**
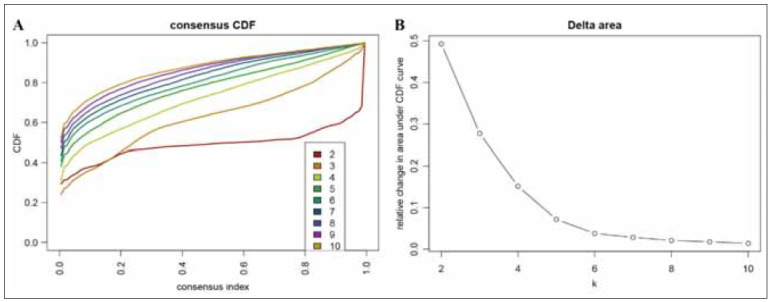
DNA methylation cluster analysis. (A) Consensus CDF curve categories of DNA methylation. (B) Delta area of relative change in area under CDF curve.

The 8161 CpG sites alone linked to the prognosis of EC were subjected to analyzed using consensus clustering. This approach aimed to identify distinct DNA methylation. The consensus cluster result showed that the consensus cumulative distribution function (CDF) curve started to steady following the formation of class 6 or 7 (Figure 1A). We chose 6 subgroups because it led to a rising inflection point of the curve (Figure 1A-1B).

### DNA methylation subtype identification

**Figure 2:**
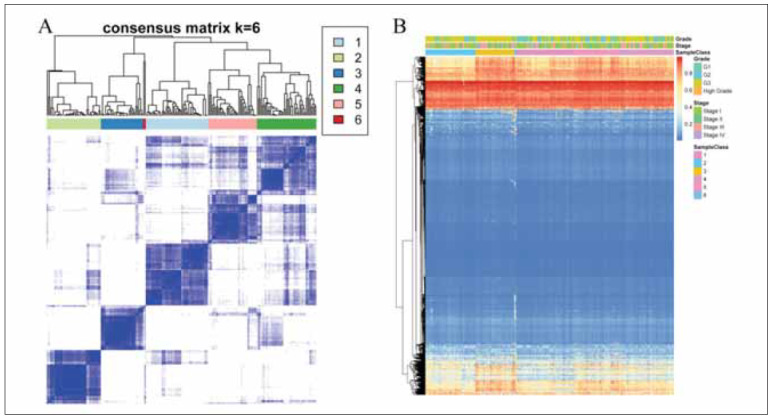
DNA methylation cluster analysis. (A) Consensus matrix heatmap plot of DNA methylation subgroups. (B) Heatmap plot of CpGs in different subgroups.

By performing consistent clustering on these cases in the training set, a heatmap characterized by featuring blue blocks positioned diagonally on a canvas of white color was obtained, which represented the consensus for k=6 (Figure 2A). The heatmap is consistent with the dendrogram with stage, grade, DNA methylation classification, and age as the annotations (Figure 2B). Notably, the six subgroups displayed distinct methylation expression profiles, with cluster 3 demonstrating a notably higher methylation level compared to the others.

### DNA methylation subtype clinical characteristic

**Figure 3:**
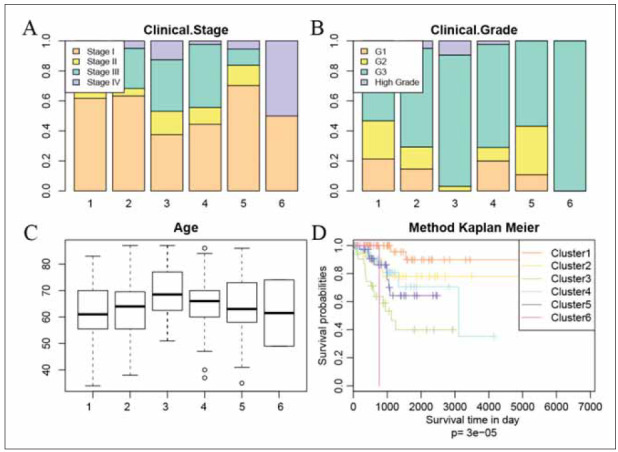
Clinical characteristic in different subgroups. (A) The clinical stage ratio in different subgroups. (B) The clinical grade ratio in different subgroups. (C) The age distribution in different subgroups. (D) The K-M plot in different subgroups.

Subsequently, we conducted an assessment of the prognostic outcomes across the nine clusters. A log – rank test was carried out among these subgroups. Our findings indicated that the cluster 1 has the best prognosis, the cluster 3 has poor prognosis. This result indicated that the patient who has a high DNA methylation level may have a poor prognosis than the low. Interestingly, we found that the patients in advanced stage and Grade3 also correlated with poor prognosis (Figure 3A and Figure 3B). There is no significant difference between the age among 6 clusters (Figure 3C). Kaplan–Meier plot showed that the cluster3, cluster4 and cluster5 had a poor prognosis than others (Figure 3D).

### CpGs annotation and enrichment analysis

**Figure 4:**
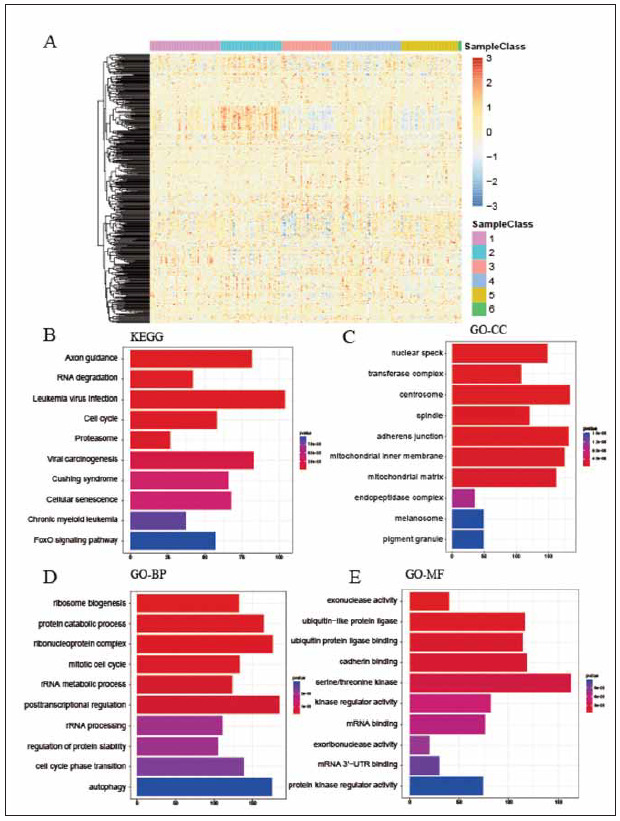
CpGs annotation and enrichment analysis. (A) Heatmap plot of DNA methylation annotation in different subgroups. (B-E) KEGG and GO enrichment analysis in different subgroups.

As a result, there are 6946 genes annotation by 8161 CpGs corresponding to the promoter regions(Figure 4A). The top 10 KEGG pathways were: Axon guidance, RNA degradation, Leukemia virus infection, Cell cycle, Proteasome, Viral carcinogenesis, chronic myeloid leukemia, Cushing syndrome, Cellular senescence and FOXO signaling pathway (Figure 4B). GO enrichment analysis exhibited that the varied genes were related to ribosome biogenesis, ubiquitin−like protein ligase and nuclear speck (Figure 4C-4E).

### WGCNA analysis

**Figure 5:**
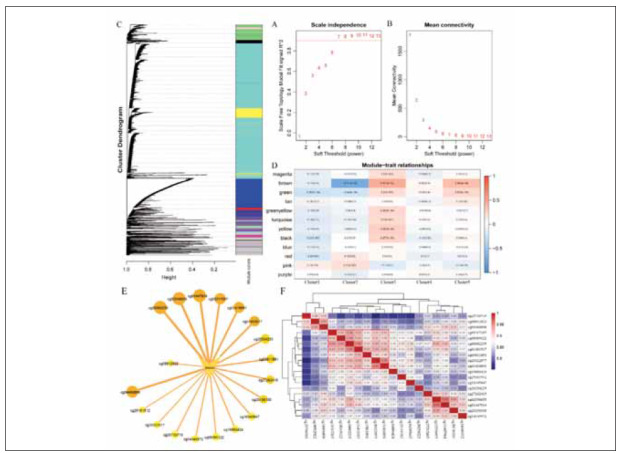
WCGNA analysis. (A-D) WCGNA analysis between DNA methylation level and prognosis. (E-F) PPI network identified key target and its relevance in WCGNA analysis.

This study used the WGCNA algorithm to construct a co-expression network based on these 8161CpGs by WGANA package. The scale independence plot showed that the best soft threshold was 7 (Figure 5A-5B). At last, the WGCNA analysis identified 12 modules and the gray module was excluded (Figure 5C). Cluster3 was positively correlated with lots of the modules, and Cluster1 was negatively correlated with lots of the modules. The brown module exhibited a positive correlation with Cluster3. Conversely, it displayed a notably negative correlation with both Cluster2 and Cluster1. We select the CpGs in the brown module and screen them based on their correlation with the module CpGs with a correlation coefficient greater than 0.9 (Figure 5D). Further analysis between these 19 CpG showed that cg20322977, cg01416891, cg00082235, cg01493517, cg08395122, cg03811891, and cg05317207 was the correlated with DNA methylation (Figure 5E). Then, the five CpG were annotated in RP11-348J12.2, IFFO1, CACYBPP3 and CTD-2619J13.8 (Table 2). At last, we chose the intersection of the top 10 most relevant to the brown module and the 5 most relevant CpGs as the feature CpGs (Figure 5F).

**Table 2:** 5 CpG annotation.

CpG	Chrom	Start	End	GeneSymbol
cg01416891	chr10	93061135	93061136	RP11-348J12.2
cg00082235	chr12	6555370	6555371	IFFO1
cg01493517	chr12	6556281	6556282	IFFO1
cg03811891	chr6	1.59E+08	1.59E+08	CACYBPP3
cg05317207	chr19	58356303	58356304	CTD-2619J13.8

### Methylation-related gene panel construction and validation

**Figure 6:**
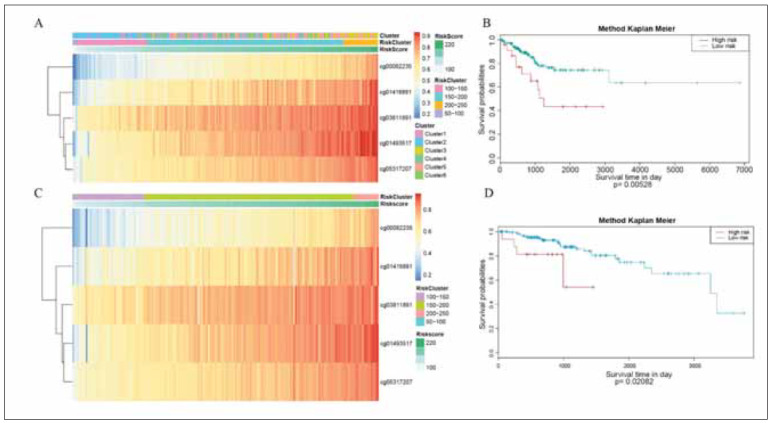
Gene panel construction and validation. (A-B) gene panel in training set. (C-D) gene panel in validation set.

We used the 5 CpGs detected by WGCNA to construct a prognosis gene panel. We divided the patients into Low-risk group (50-100 risk score), Middle-risk group (100-200 risk score) and High-risk group (200-250 risk score) and found that the prognosis of the high-risk group was poor (Figure 6A-6D). Next, we used the validation set to test the accuracy of prognosis gene panel. The result showed that the prognosis gene panel had a good prediction ability in EC prognosis.

## Discussion

EC represents a malignant tumor, which has high incidence and mortality [18]. It manifests indiverse molecular patterns and histological subtypes, including type II (high-grade) and type I (low-grade). In 2014, a research showed that EC could divided into four subtypes thought copy number alterations, microsatellite instability (MSI) and PLOE mutation [19]. However, there was no methylation subtype in EC. In our study, we found that 8161 CpGs were hypermethylation in EC patients. After that, we used unsupervised clustering algorithm to identify different subgroup in feature CpGs and found that cluster3, cluster4 and cluster5 had a poor prognosis than other clusters. Eventually, CpGs cg01416891, cg00082235, cg01493517, cg03811891 and cg05317207 were closely correlated with EC prognosis by using WGCNA. In our study, we found that the top 10KEGG pathways were: Axon guidance, RNA degradation, Leukemia virus infection, Cell cycle, Proteasome, Viral carcinogenesis, chronic myeloid leukemia, Cushing syndrome, Cellular senescence and FOXO signaling pathway. While some of these pathways, such as the Cell cycle [20], Proteasome [21], Cellular senescence [22], and FOXO signaling pathway [23], have well-documented roles in cancer pathogenesis and progression, others may have less obvious connections to the typical pathogenic mechanisms of EC. However, the complexity of cancerpathogenesis and context-dependent roles of biological pathways suggest that even seemingly unrelated pathways could play important roles in specific contexts or subtypes of EC. The GO enrichment analysis exhibited that the varied genes were related to ribosome biogenesis, ubiquitin−like protein ligase and nuclear speck. In order to evaluate the accuracy, the CpGs for prediction of EC prognosis, the five CpGs were constructed a prediction model. The result showed that both training set and validation set had a good performance in prediction of EC overall survival.

Previous study showed that the prediction model contain methylation genes of CDH13, GHSR and SST had a good performance in prediction of prognosis in EC patients [24]. The other research indicated that PTEN, a tumor suppressor gene, has a decreased expression level in EC tissues due to its promoter hypermethylation [25]. In the other hand, urine methylation markers of EC patients (GHSR, SST and ZIC1) could be a novel method to detect EC [26]. However, there was no research that RP11-348J12.2, IFFO1, CACYBPP3 and CTD-2619J13.8 in EC patients. Our study showed that the five CpGs would be a new site of methylation in prediction of EC patients. These four methylation genes might be a potential marker in EC.

Nowadays, conventional EC diagnosis encounters many limitations and challenges. TVS remainshas a low sufficient in distinguishing benign and malignant endometrial lesions, displaying low characteristic among potential patients [27]. How to improve the accuracy and specificity in EC diagnosis still a problem. Our study indicated that this five DNA methylation characteristic could identify the EC subtype and had a good performance in prediction of EC prognosis. In the future, the specificity of this approach would provide a new sight, encompassing both asymptomatic and symptomatic women who are at risk of EC, as well as those with benign endometrial conditions.

While our study has provided valuable insights into the potential role of DNA methylation in distinguishing between low-risk and high-risk groups, it is important to acknowledge several limitations. Firstly, our analysis is primarily based on bioinformatics and statistical methods, which, although powerful, require experimental validation to confirm the biological relevance of the identified methylation sites. Secondly, the specific functional roles of the key methylation sites identified in our study remain to be elucidated. Future research should focus on conducting in vitro and/or in vivo experiments to verify the function of these methylation sites and to investigate their impact on gene expression, cellular behavior, and ultimately, patient outcomes.

## Conclusion

The methylation-related gene panel model, in - cor porating cg01416891, cg00082235, cg01493517, cg03811891, and cg05317207, demonstrates robust prognostic value in EC. Patients classified as high-risk based on this model exhibit significantly poorer outcomes compared to low-risk counterparts, underscoring its potential as a reliable biomarker for predicting EC prognosis and guiding personalized treatment approaches.

## Dodatak

### Declarations

#### Ethics approval and consent to participate

Not applicable.

### Consent for publication

Not applicable.

### Availability of data and materials

RNA-seq data and clinical information contain Age, Stage and Grade for 409 EC samples were downloaded from the TCGA database (https://www.cancer.gov/ccg/research/genome-sequencing/tcga).

### Funding

None.

### Authors’ contributions

Conceptualization and design, Yue Pang and Pu Cheng; Provision of study materials or patients, Junyan Li, Jiong Ma; Collection and assembly of data, and Data analysis and interpretation Lijuan Jiao; Final approval of manuscript: All authors; Manuscript writing: All authors.

### Acknowledgements

The authors express their appreciation to staff in The Second Affiliated Hospital Zhejiang University School of Medicine, for their technical assistance.

### Conflict of interest statement

All the authors declare that they have no conflict of interest in this work.
